# Indirect Evidence Link PCB Dehalogenation with Geobacteraceae in Anaerobic Sediment-Free Microcosms

**DOI:** 10.3389/fmicb.2016.00933

**Published:** 2016-06-16

**Authors:** Martina Praveckova, Maria V. Brennerova, Christof Holliger, Felippe De Alencastro, Pierre Rossi

**Affiliations:** ^1^Laboratory of Molecular Genetics of Bacteria, Institute of Microbiology, Academy of Sciences of the Czech RepublicPrague, Czech Republic; ^2^Faculty of Science, Charles University PraguePrague, Czech Republic; ^3^Laboratory for Environmental Biotechnology, School of Architecture, Civil and Environmental Engineering, Ecole Polytechnique Fédérale de LausanneLausanne, Switzerland; ^4^Central Environmental Laboratory, School of Architecture, Civil and Environmental Engineering, Ecole Polytechnique Fédérale de LausanneLausanne, Switzerland

**Keywords:** polychlorinated biphenyl congeners, Delor 103, reductive dechlorination, sediment-free microcosms, *Dehalococcoides*, Geobacteraceae

## Abstract

Although polychlorinated biphenyls (PCBs) production was brought to a halt 30 years ago, recalcitrance to degradation makes them a major environmental pollutant at a global scale. Previous studies confirmed that organohalide-respiring bacteria (OHRB) were capable of utilizing chlorinated congeners as electron acceptor. OHRB belonging to the Phyla *Chloroflexi* and *Firmicutes* are nowadays considered as the main PCB-dechlorinating organisms. In this study, we aimed at exploring the involvement of other taxa in PCB dechlorination using sediment-free microcosms (SFMs) and the Delor PCB mixture. High rates of congener dehalogenation (up to 96%) were attained in long-term incubations of up to 692 days. Bacterial communities were dominated by *Chloroflexi, Proteobacteria*, and *Firmicutes*, among strictly simplified community structures composed of 12 major phyla only. In a first batch of SFMs, *Dehalococcoides mccartyi* closely affiliated with strains CG4 and CBDB1 was considered as the main actor associated with congener dehalogenation. Addition of 2-bromoethanesulfonate (BES), a known inhibitor of methanogenic activity in a second batch of SFMs had an adverse effect on the abundance of *Dehalococcoides* sp. Only two sequences affiliated to this Genus could be detected in two (out of six) BES-treated SFMs, contributing to a mere 0.04% of the communities. BES-treated SFMs showed very different community structures, especially in the contributions of organisms involved in fermentation and syntrophic activities. Indirect evidence provided by both statistical and phylogenetic analysis validated the implication of a new cluster of actors, distantly affiliated with the Family Geobacteraceae (Phylum δ-*Proteobacteria*), in the dehalogenation of low chlorinated PCB congeners. Members of this Family are known already for their dehalogenation capacity of chlorinated solvents. As a result, the present study widens the knowledge for the phylogenetic reservoir of indigenous PCB dechlorinating taxa.

## Introduction

Polychlorinated biphenyls (PCBs) have been used massively in diverse industrial applications due to their extreme resistance to high temperature and their high chemical stability. Hundreds of thousands of metric tons of commercial PCB congener mixtures have been manufactured from 1929 to the ban of their production (US EPA, 2005). PCB congeners released worldwide bio-magnify along the food chain and exert multiple adverse health effects. Brown et al. linked for the first time the observed weathering patterns of the congeners *in situ* with microbial degradation activities (Brown et al., 1988). These findings paved the way to extensive laboratory studies showing that anaerobic stepwise dechlorination by specific organohalide-respiring bacteria (OHRB) was the prerequisite key process toward their complete mineralization by aerobic bacteria (Bedard et al., 2007; Field and Sierra-Alvarez, 2008; Passatore et al., 2014). Occurrence of this dehalogenation mechanism was shown to be widespread, making it the dominant removal process affecting the fate of PCB congeners in natural and man-made environments (Rodenburg et al., 2010).

However, and despite numerous studies carried out so far, limited information is available about the diversity and the ecology of the OHRB guild members participating to the dechlorination process (Yan et al., 2006; Bedard, 2008; Hiraishi, 2008; Kjellerup et al., 2012). *Dehalococcoides mccartyi* strain CBDB1 (Adrian et al., 2009), strain 195 (Fennell et al., 2004), and strain JNA (LaRoe et al., 2014), *Dehalobium chlorocoercia* DF-1, *bacterium* o-17 (Cutter et al., 2001), *Dehalogenimonas lykanthroporepellens* (Wang and He, 2013b), and finally the phylotypes m-1, SF-1, and DEH10 (Fagervold et al., 2007) demonstrated PCB-dechlorinating capacities. These organisms form a monophyletic clade, deeply branching within the *Chloroflexi* phylum. Recently, Wang et al. identified for the first time the PCB reductive dehalogenases present in three *Dehalococcoides* strains (Wang et al., 2014). Each of these enzymes was shown to reduce dozens of different PCB congeners specifically and leading to different end products (Bedard, 2014). These results confirmed preceding results showing that members of the *Chloroflexi* phylum exhibited specifically a limited range of PCB congener dechlorination capacity (Hiraishi, 2008). The distribution of genes targeting different congeners within distinct organisms strongly suggests the existence of synergistic mechanisms within the OHRB guild and organisms far more diversified than expected initially. The only exceptions to the dominance of the *Chloroflexi* OHRB reported to date are the phylotypes FTH1 and FTH2 closely related to *Dehalobacter* sp. among the Phylum *Firmicutes* (Oh et al., 2008; Yoshida et al., 2009; Wang and He, 2013a). Early results provided by Ye et al. demonstrated the implication of sulfate-reducing, spore-forming organisms in pasteurized dechlorinating cultures (Ye et al., 1992). This line of research was discontinued nonetheless, despite clear evidences of the presence of members of the *Firmicutes*, the only Phylum including sporulating, sulfate-reducing organisms. Other lines of research showed that OHRB from the Phylum *Proteobacteria* and *Bacteroidetes* may be involved equally (May et al., 2008). Members of the so-called facultative OHRB, such as *Anaeromyxobacter, Desulfitobacterium, Sulfurospirillum, Desulfomonile, Desulfuromonas, Desulfovibrio*, and *Geobacter* were shown to dechlorinate a wide range of halogenated alkane, alkene and cyclic hydrocarbons. However, their potential capacity for PCB dechlorination has not been demonstrated with certainty yet. These Genera are nonetheless very flexible in terms of catabolic reactions and, as sulfate or sulfite reducers, their presence was shown to be positively influencing OHR activities of bacteria involved in the dechlorination of PCB congeners in enrichment cultures (Zwiernik et al., 1998; Wu et al., 2002). The role of *Archaea* in PCB dehalogenation is still unclear despite the recent findings of a putative reductive dehalogenase linked with the *Euryarchaeota* (Hug et al., 2013). Ye et al. reported the possible, albeit restricted, dechlorination activity carried out by methanogens (Ye et al., 1995), a hypothesis which was refuted by the same authors in later reports based on the results obtained from experiments carried out using pasteurized microbial communities (Ye et al., 1999). Other studies (Oh et al., 2008) showed that *Archaea*, and especially acetoclastic methanogens, may play an indirect role in PCB dechlorination in the synthesis of cobalamin, a corrinoid cofactor required for vitamin B12 synthesis, which is necessary for assembling functional reductive dehalogenase systems (Rupakula et al., 2015). Cobalamine and other precursors are typically produced by methanogenic Archaea as cofactors for methyltransferases involved in methane formation (Yan et al., 2013).

The Strazske River derivation channel (Slovak Republic) received the effluents of the largest PCB factory in former Czechoslovakia until the cease of production in 1984 (Dercová et al., 2008). The factory manufactured ca. 60% of the whole production in Eastern Europe (Taniyasu et al., 2003). Very high concentrations of PCB congeners (up to 146 mg/kg) persisted in the river sediment 25 years after the cease of production making this zone one of the most heavily PCBs-contaminated areas worldwide (Praveckova et al., 2015). Results from a PCB congener dehalogenation experiment using sediments from the Strazske River showed the implication of predominant OHRB affiliated with *Dehalococcoides* sp., as well as unknown deep branching clades among the Class *Dehalococcoidia* (Praveckova et al., 2015). Dudková et al., working on the same location, reported that PCB dechlorination activity was novel and did not correspond to any of the previously identified microbial PCB dechlorination processes or to any combination thereof (Dudková et al., 2012). The goals of the present study were to identify the OHRB putatively involved in the dehalogenation observed so far. Simplified community structures were targeted using sediment-free microcosms (SFMs) and Delor congeners, inoculated with the supernatant from the first generation of sediment microcosms developed previously (Praveckova et al., 2015). As a recall, SFMs have been used for the analysis of the dehalogenation patterns and involved OHR bacterial guild members (Cutter et al., 1998; Bedard et al., 2006, 2007; Dudková et al., 2012). The advantages of SFMs over traditional enrichments are multiple and include the standardization of the cultures, uniform sampling, as well as the strong decrease of any influence from residual concentrations of undesired compounds. 2-bromoethanesulfonate (BES) was added in selected SFMs to inhibit methanogenic Archaea. The specific objectives of this research were (i) to characterize in detail the communities involved in the congener dehalogenation using massive sequencing techniques, (ii) to identify putative congener dehalogenation actors by combining phylogenetic information and statistical analysis, and (iii) to assess the impact of BES on the communities and the concomitant PCB dehalogenation capability.

## Materials and methods

### Anaerobic sediment-free microcosm

Fourteen anaerobic SFMs were set up according to the technique developed by Bedard et al. (2006). Delor 103 congener mixture (Chemko Strazske, Slovakia) was added as a concentrated acetone solution to silica particles (ca. 240 mesh). An aliquot of 300 mg of particles was added to a sterile glass bottle and the acetone was evaporated. One mL of acetone solution containing 2,6-dibromobiphenyl (final concentration 350 μM) was added as priming molecules and was evaporated, as shown in Dudková et al. (2012). Two hundred mL of *Dehalobacter* sp. liquid growth medium (Holliger et al., 1998) was added aseptically, providing a final PCB congener concentration of 20 mg/L. This cultivation medium contained 0.5 mg/L Resazurin (7-hydroxy-10-oxidophenoxazin-10-ium-3-one) used as an indicator of possible oxygen intrusion. Two mL of a 1:1:1:1 mixture of 100 mM (each) ethanol:propionate:butyrate:acetate were added initially as electron and carbon sources. Every 6 weeks, additional 4 mL of this mixture were added. The gaseous phase was exchanged aseptically with N_2_:CO_2_ (80%:20%; v/v) keeping overpressure insuring total anaerobic conditions. Six SFMs received 2-Bromoethanesulfonate (BES) (final concentration 5 mM), a selective inhibitor of methanogenesis (Ye et al., 1999). All SFMs were inoculated with 20 mL of the supernatant from the 12 month-old sediment microcosm M1, in which a congener dehalogenation of ca. 6.26% was measured (Praveckova et al., 2015). Microcosm M1 was inoculated with sediment samples from the Strazske derivation channel (Eastern Slovakia), massively contaminated by weathered PCB congeners. SFMs were incubated in the dark at 30°C, without agitation. Overpressure inside the microcosms was measured on a regular basis using a pressure transductor and was kept between 0.2 and 0.6 bar during the whole duration of the operation. Control SFMs were composed of both autoclaved (killed) microcosms and non-inoculated microcosms.

### PCB congener analysis

Analysis of the congeners was carried out in acetone and hexane-washed glassware, with the concomitant preparation of a glassware-blank. Internal extraction standard PCB 189 (35 μg/mL in acetone) was added to all samples prior to the extraction. Samples composed of the entire SFM volume (ca. 150 mL, after sampling for molecular analysis) were extracted twice with hexane. The volume of the extracts was reduced to 2–3 mL by rotating evaporator (40°C at 330 mbar). Samples were diluted 8.5x in isooctane and 1 μL was injected on an Agilent 6890 gas chromatograph equipped with an Agilent automatic injector and μECD detector. Delor 103 mixture (20 mg/L) was used as a standard. Separation was carried out using a DB-5 ms type capillary column (60 m × 0.25 mm I.D., df: 0.25 μm). Helium was used as carrier gas at a constant pressure of 35 psi. Injections were carried out in on-column mode, with the following temperature program: 85°C, 0.2 min hold and 70°C/min to 250°C. The GC oven was programmed as follows: 80°C, 0.5 min hold, 50°C/min to 150°C, 1 min hold, 2.5°C/min to 285°C, 30 min hold (total time: 86.9 min.). The ECD was set at 300°C. Identification of 74 PCBs congeners was carried out according to the retention time using both congener standards and literature references (Frame et al., 1996; Taniyasu et al., 2003). Delor 103 (Chemko Strazske, Slovakia) was used as a calibration sample for the correction of possible shifts in retention times. Computation of the concentration of the congeners was carried out using the detailed analysis of the Delor 103 mixture presented by Taniyasu et al. (2003) and Grabic et al. (2006). A total of 74 congeners (out of 98 in the original mixture) were quantified, including all major contributors. The IUPAC numbering system was used to designate each congener.

### DNA and RNA extractions

DNA extraction was carried out using ca. 15 mL of the SFM contents (SFM2, 3, 12, and 14) as well as 15 mL of the original sediment microcosm M1, withdrawn using a sterile syringe. Both silica particles and bacterial cells were harvested by centrifugation (4500 × g, 10 min, room temperature). The pellet was re-suspended in 400 μL of TE buffer and 100 μL of lysozyme (25 mg/mL, Sigma-Aldrich, Germany) was added to disrupt the cell walls. Samples were incubated at 37°C for 1 h and transferred in a 2 mL screw-cap tube followed by the addition of 1 mL of the extraction buffer (5 M Guanidine thiocyanate, 100 mM EDTA, 1% Na-lauroyl sarcosinate, 1% PVP K30, 150 mM sodium phosphate buffer pH = 8.0, all AppliChem, Germany) as well as by 0.5 g of 0.1 mm diam. sterile zirconia-silica beads (Thermo Fisher Scientific, USA) and by 5 μL of a 1 M DTT solution (AppliChem, Germany). The tubes were incubated at 60°C for 5 min. and processed in a FastPrep FP120 Cell Disrupter (Bio101, USA) for 30 s at 4.5 m/s. The tubes were centrifuged at 12,000 × g for 2 min and 1 mL of the supernatant was added to a SEV DNA Tissue Extraction Kit cartridge (Promega, USA). Cartridges were processed according to the manufacturer instructions in an automated Maxwell® 16 Research System (Promega, USA). The purified DNA was recovered in 200 μL of 10 mM Tris HCl pH 7.5 buffer.

For total RNA extraction, 50 mL of the SFMs and M1 microcosm supernatant were harvested by centrifugation (4500 × g, 10 min, room temperature). The pellet was re-suspended in 1 mL of Stop RNAse Solution (Qiagen, Germany) and was transferred to a 1.5-mL microcentrifuge tube and centrifuged (12,000 × g, 1 min, room temperature). Supernatant was discarded and the pellet was either frozen in liquid nitrogen and stored at −80°C or extracted immediately. The pellet was re-suspended in 100 μL of DNAse and RNAse-free TE buffer containing 25 mg/mL lysozyme and incubated for 37°C for 1 h. Total RNA was extracted using the SV Total RNA Isolation System (Promega, USA) according to the manufacturer instructions. An additional DNAse digestion step was carried out so as to remove all traces of undesired carry-over material. To do so, 28 μL of the extracted RNA were incubated with 2 μL of DNAse and RNAse-free water, 3.75 μL of RQ RNAse-free DNAse 10x reaction buffer and 3.75 μL of RQ RNAse-free DNAse (1 U/μL) for 30 min at 37°C. Subsequently, DNAse was inactivated by the addition of 3.75 μL DNAse Stop Solution (all Promega, USA) for 10 min at 65°C and kept on ice. 2 μL of random hexanucleotides (0.5 μg/μL) (Microsynth, Switzerland) were incubated with 18 μL of the DNAse-free RNA samples for 5 min at 70°C and kept on ice until further processing. cDNA was obtained with the GoScript reverse transcriptase according to the manufacturer instructions (Promega, USA). Reverse transcription reaction was conducted in a Biometra T3000 thermal cycler (Biometra, Germany) as follows: annealing step at 25°C for 5 min, elongation 42°C for 90 min and inactivation 70°C for 15 min.

### Bacterial community analysis

Amplification of the variable regions V1-V3 of 16S rRNA gene was carried out on cDNA and DNA samples using the primers 28f (GAGTTTGATCNTGGCTCAG) and 519r (GTNTTACNGCGGCKGCTG). 50 μL PCR reactions containing 5 ng template were carried out as follows: 10 μL 5X KAPA HiFi Fidelity Buffer (Kapa Biosystems, USA), 1.5 μL of 10 mM dNTPs, 1 U KAPA HiFi Polymerase (Kapa Biosystems, USA), 1.5 μL of both primers at 10 μM. Amplifications were conducted in a T3000 Thermal Cycler (Biometra, Germany) with an initial denaturing step at 95°C for 4.5 min, followed by 25 cycles of denaturation (98°C for 20 s), annealing (56°C for 15 s) and extension (72°C for 30 s), followed by a final extension step (72°C for 5 min). Amplicons were sequenced on a Roche GS-FLX+ instrument (Roche, Switzerland) by Research and Testing Laboratory (USA). Sff files were processed using Mothur (Schloss et al., 2009) on a Biolinux 7 platform as described in Diaby et al. (2015), providing between 1193 (original sample M1) and 6282 final reads (SFM7).

The freeware R (Team, 2014) and the Vegan package (Oksanen et al., 2013) were used for numerical ecology analysis such as Principal Component Analysis (PCA) and Multifactorial analysis (MFA). MFA is a generalization of PCA allowing the analysis of several data sets collected on the same set of observations and evaluates the possible presence of relationships between variables or groups of variables. MFA explored the correlations between bacterial Families and classes of congeners, as well as the correlations between environmental parameters themselves. R was used equally for inference statistics, for the computation of Fisher's α index (a semi-parametric index independent from the size of the sampling) and Pielou's evenness. Hierarchical clustering was carried out using Esprit-Tree (Cai and Sun, 2011). The output was used to compute Operational Taxonomic Units (OTUs), rarefaction curves, as well as Abundance-based Coverage Estimators (ACE) and CHAO1 estimators, which assessed the putative total richness by adding a correction factor to the observed number of species.

Sequences obtained in this study were sent to the Sequence Read Archive (SRA) at NCBI under the BioProject ID PRJNA315726.

## Results

### Dehalogenation of the PCB congeners

Fourteen SFMs were amended with Delor congeners and inoculated with PCB-degrading bacterial communities from a sediment microcosm M1 described previously (Praveckova et al., 2015). SFMs were incubated up to 692 days with regular additions of electron donors. No cultivation transfer was required for the development of stable active microbial communities showing efficient dehalogenation capacities on the long term. The dehalogenation patterns of the congeners observed in all SFMs are summarized in Table 1. Detailed results of the congener distributions are shown in the Table SI-1. GC-μECD analysis showed that the 2,6-dibromobiphenyl added for the initial priming of the dehalogenation reactions was fully degraded and could not be detected in the first samples analyzed at day 98 (data not shown). No apparent inhibition of the priming solution could be observed on the functioning of the SFMs, as it was reported in former studies (Bedard et al., 2006).

**Table 1:** **Dehalogenation patterns of 74 congeners present within the Delor 103 mixture observed in the SFMs**.

**SFM**	**No BES**								**BES added**							
**Days^a^**	**1**	**2**	**3**	**4**	**5**	**6**	**7**	**8**	**9**	**10**	**11**	**12**	**13**	**14**	**CTRL^b^**	**REF^c^**
	**98**	**126**	**126**	**155**	**173**	**173**	**569**	**692**	**98**	**121**	**126**	**126**	**569**	**692**	**503**	
Di-CB	1.63	2.31	0.79	1.10	2.50	3.77	0.00	1.29	0.66	3.29	0.39	0.20	0.00	0.00	1.7 (11.2%)	10.4%
Tri-CB	11.80	9.70	9.88	8.82	7.75	10.28	0.38	4.96	9.09	12.00	7.50	10.43	0.38	2.29	9.9 (58.0%)	53.8%
Tetra-CB	5.54	4.05	4.17	5.12	4.50	4.95	0.37	2.70	4.57	4.20	3.07	4.83	0.30	2.20	5.6 (28.8%)	32.3%
Penta-CB	0.27	0.19	0.20	0.28	0.24	0.29	0.04	0.27	0.25	0.19	0.15	0.25	0.15	0.29	0.4 (1.7%)	2.6%
Hexa-CB	0.05	0.03	0.03	0.05	0.02	0.05	0.00	0.07	0.04	0.03	0.03	0.04	0.05	0.07	0.1 (0.3%)	0.3%
Hepta-CB	0.01	0.00	0.00	0.01	0.00	0.01	0.00	0.01	0.01	0.00	0.00	0.01	0.01	0.01	0.0 (0.1%)	0.1%
TOTAL^d^	0.0	7.7	14.5	12.8	14.9	0.0	95.5	47.3	17.2	0.0	36.9	10.7	94.9	72.5		

*Remaining PCB congeners sharing the same amount of chlorine atoms are expressed in mg/L*.

a Number of days of continuous cultivation;

b Killed (autoclaved) control, expressed both in mg/L and, in parenthesis, in Mole%;

c Original Delor 103 mixture in Mole% according to Grabic et al. (2006);

d *Fraction (in %) of total PCB congeners removed compared to the control*.

Chemical analysis of 74 congeners showed that dehalogenation occurred in all SFMs and that all congener classes were impacted by the dehalogenation (Table 1). Di- and Tri-CB were the only classes showing significant transitional accumulation of congeners produced by the reduction of congeners (Table SI-1). Highest dechlorination rates were observed in the oldest SFMs, at 569 and 692 days. At day 569, 95.5% and 94.9% of all congeners were removed in SFM7 and in SFM13 respectively. SFMs 1, 5, 6, and 10 showed an activity of dechlorination, but the total amount of congener molecules was kept constant, with the accumulation of Di- and Tri-CB. Despite the inoculation of all SFMs with identical inoculum, discrepancies could be observed for the duplicate SFMs analyzed on the same day of cultivation, as for instance for SFM5 and SFM6 (day 173, without BES), and SFM11 and SFM12 (day 126, with BES). As shown in Table 1, no significant difference could be observed when BES was added to the SFMs in terms of degradation rates.

### Microbial community structures

Massive sequencing data from the genetic pool of the 12 16S rRNA gene amplicons resulted on average in 6294 reads, ranging from 2567 to 9695 sequences. After quality control steps (such as denoising and the removal of putative chimeras), subsequent analysis was carried out on a total of 49,686 sequences, ranging from 1193 (sample M1) to 6282 sequences (SFM7) (Table SI-2). Fisher's alpha showed a strong reduction of the diversity in all SFMs when compared to the sediment microcosm M1. The richest community was present in SFM12 (9.76) while the lowest value was calculated for SFM8 (6.77). The decrease of diversity from M1 to the SFMs was corroborated with the computation of the number of observed Genera, passing from 152 (M1) to maximum of 96 (SFM1) (Figure SI-1). However, this reduction was not equal for all SFMs despite the homogenization of the culture conditions. Statistical analysis showed no significant impact of BES on the apparent total richness, diversity and evenness of the communities computed at the Genus level, as well as the amount of time of incubation (Table SI-2). Although no significant trends could be observed at the bacterial phylum level (Table 2), the addition of BES had clear consequences on the lower bacterial taxonomic levels (Table SI-4), illustrating niche differentiation between deeply diverging phylogenetic lineages. The addition of the inhibitor particularly affected the relative proportions of taxa composing the Phyla *Proteobacteria, Chloroflexi*, and *Firmicutes*.

**Table 2:** **Relative contributions (in %) of the main Phyla present in the SFMs, obtained from the analysis of total RNA. “DNA” indicates that communities were analyzed from total DNA extracted in parallel from the same SFM**.

		**No BES**										**BES added**							
**Phylum**	**M1-DNA**	**SFM1**	**SFM2**	**2-DNA**	**SMF3**	**3-DNA**	**SFM4**	**SFM5**	**SFM6**	**SFM7**	**SFM8**	**SFM9**	**SFM10**	**SFM11**	**SFM12**	**12-DNA**	**SFM13**	**SFM14**	**14-DNA**
*Actinobacteria*	1.26	0.06	0.17	0.74	0.03	0.05	0.02	0	0.11	0	0.12	1.05	0	0.12	0.22	7.41	0.52	0.26	6.28
*Bacteroidetes*	0.76	0.60	1.11	2.78	3.08	2.70	5.75	0.28	0.58	0.10	0.16	1.59	5.08	1.10	2.66	8.39	0.72	1.87	1.05
*Chloroflexi*	0.76	67.06	35.30	17.48	42.55	5.29	74.06	2.90	22.66	6.92	7.87	48.18	17.32	33.65	57.67	28.20	8.49	4.88	18.16
*Firmicutes*	18.81	3.45	1.67	45.70	9.12	52.78	1.27	46.96	8.61	30.47	45.90	0.88	3.68	2.42	2.84	22.90	1.32	8.04	32.99
*OP3*	0.08	0	0	2.22	12.73	10.84	0.02	0	0.21	0	0	0	0	0	0	0	0	0	0
*Planctomycetes*	0	0.56	0.10	0.51	0.21	0	0.03	0.06	0.30	10.62	2.54	0.41	0.37	0.02	0.04	0.12	0.32	5.29	0.13
*Proteobacteria*	57.18	15.81	58.76	9.62	2.76	1.69	10.39	35.23	64.08	33.21	38.03	18.97	49.25	58.21	29.89	15.11	85.30	70.24	37.55
*Spirochaetes*	0.59	0.89	0.07	0.46	0.39	0.26	0.03	0.06	0.06	0.05	0	2.41	1.88	0.17	0.58	0.72	0.40	0.13	0.13
*Synergistetes*	0	3.84	0.52	13.92	3.87	7.72	0.27	9.33	0.64	13.51	1.53	5.50	13.35	0.72	0.85	12.57	1.04	1.85	1.31
*TM7*	7.39	0	0	0	0	0	0	0	0	0	0	0	0	0	0	0	0	0	0
*Thermotogae*	0	3.47	0.56	0.74	2.45	0.58	1.80	1.14	0.68	0.16	0.20	12.45	3.27	0.90	1.31	0.46	0.84	5.60	0.43
*WWE1*	0	0.12	0	0.09	14.46	4.60	5.45	0.11	0.13	0	0.04	5.02	0.15	0.65	1.39	0.69	0	0.33	0
Unclassified	9.91	3.74	1.67	4.90	8.31	13.33	0.88	3.93	1.90	4.97	3.51	3.19	5.52	2.01	1.94	2.97	0.88	1.51	1.90
Total	96.73	99.59	99.93	99.17	99.95	99.84	99.98	100	99.96	100	99.92	99.66	99.85	99.97	99.39	99.54	99.84	100	99.95

*The original sediment microcosm M1 is provided as a reference*.

Bacterial communities were essentially driven by 12 major Phyla only, contributing for 99–100% of the communities present in the SFMs and about 97% in the initial M1 sediment microcosm (Table 2). Among these Phyla, *Chloroflexi*, composed essentially of the classes Anaerolineae and Dehalococcoidia, *Firmicutes*, with the Class Clostridia, *Proteobacteria*, with the δ-Proteobacteria, and finally *Synergistetes*, with the Class Synergistia, were the most abundant contributors (Table SI-4). Other minor Phyla contributed marginally and sporadically to the communities, such as OP9 and *Verrucomicrobia* (Table SI-3). A few Phyla were present in the sediment microcosm M1 only, such as TM7. Other Phyla were apparently absent in the inoculum and developed in sediment-free conditions only, such as *Planctomycetes, Synergistetes*, and *Thermotogae*. The Phyla OP3 and *Planctomycetes* were found in significant proportions in a few microcosms only, such as SFM3, and always in conjunction with sequences affiliated with the Class Dehalococcoidia. Finally, relative contributions from Unclassified sequences (at the Bacteria level) were low, reaching a maximum of 8.3% in SFM3.

Comparison between RNA- and DNA-based analyses revealed strong discrepancies in the relative proportions of the Phyla *Chloroflexi* and *Firmicutes*. DNA-based analysis provided systematically lower relative contributions from members affiliated with the Family Anaerolineaceae as compared with the RNA-based analysis (Table SI-4). Inversely, analysis of the community using total DNA showed higher contributions of members affiliated with the Families Syntrophomonadaceae and Nocardioidaceae (Phylum *Actinobacteria*) in SFM14 and SFM12, showing a high prevalence of the facultative anaerobic genus *Propionicimonas* sp.

**Figure 1:**
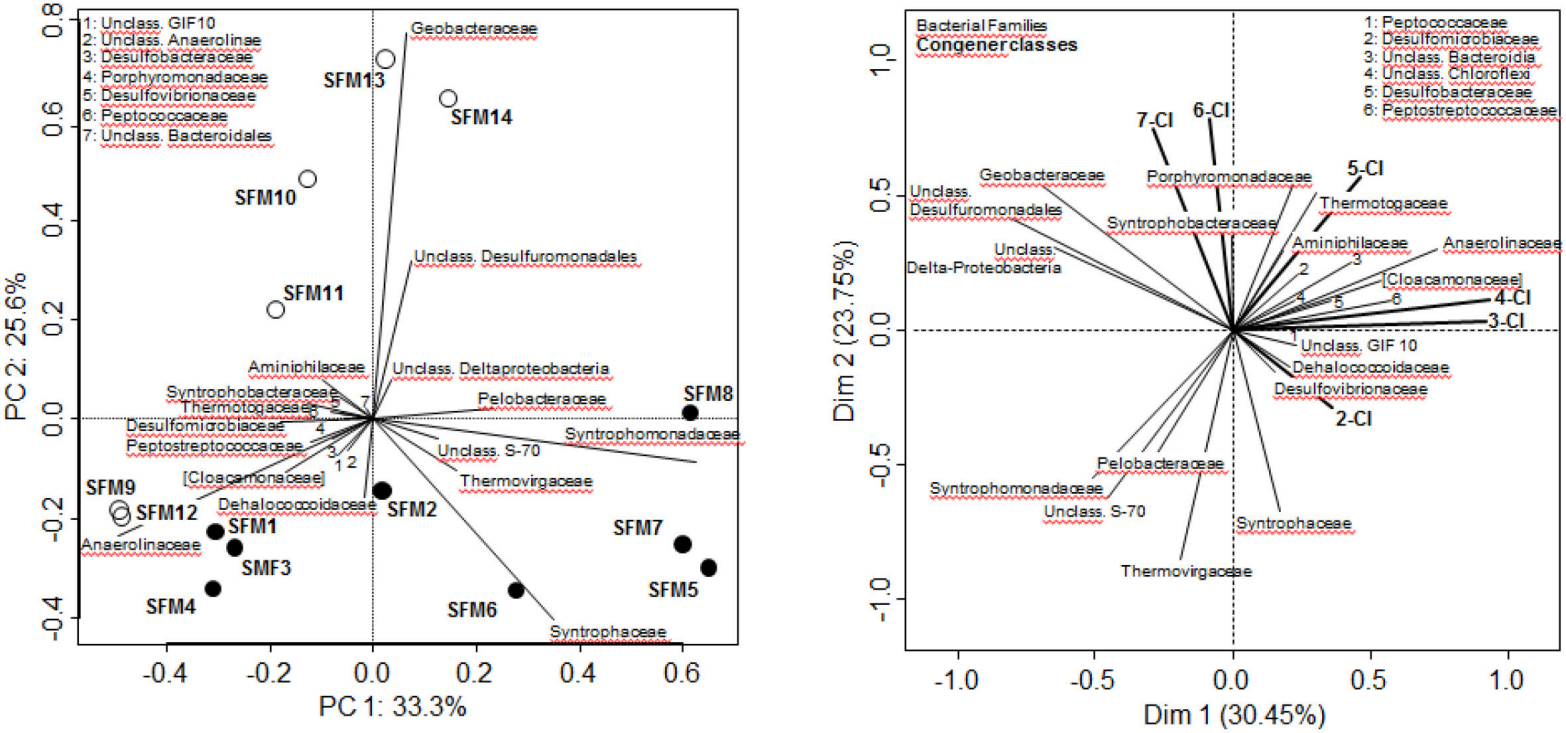
**Bacterial community analysis (RNA only) carried out at the Family level**. **Left**: Principal Component Analysis. White circles: BES-treated SFMs; black circles: SFMs without BES. **Right:** Multi-Factorial Analysis using both bacterial Families and congener classes, grouping congeners sharing the same degree of chlorination. In both analyses, only Families containing a minimum of 300 sequences (among all SFMs) were retained for the computations.

PCA analysis of whole communities at the Family level illustrated the strong disparities observed among all SFMs despite inoculation with the same microbial community (Figure 1). SFMs with short incubation times (up to 155 days) such as SFMs 1–4, 9, and 12 clustered in the lower left pane. Older SFMs showed a clear separation as a function of the addition of BES. BES-treated SFMs (open circles) migrated in the upper panes, whereas non-amended ones (black circles) clustered in the lower right pane. Up to 155 days, communities present within all SFMs were composed by strong percentages of members of the Family Anaerolineaceae. Gradually, this taxon was replaced with time in all SFMs by members of the Syntrophomonadaceae (Genus *Syntrophomonas* sp.). Older BES-treated SFMs were characterized mostly by high proportions of sequences related with taxa affiliated to the δ-Proteobacteria, such as the Families Geobacteraceae and Syntrophobacteraceae, as well as unclassified sequences linked with the Order Desulfuromonadales.

### Detailed taxonomical analysis

The search for putative taxa involved in the PCB congener dehalogenation was carried out using Multifactorial analysis (MFA), combining data sets from both bacterial diversity (expressed at the Family level) and congener classes, grouping congeners sharing the same number of chlorine atoms (Figure 1). ANOVA carried out on the MFA output showed that the congener classes could explain significantly (*p* = 0.012^*^) the distribution of the Families (data not shown). Vectors of the congener classes were distributed logically on the MFA factor map. Hepta-CB and Hexa-CB were collinear, as well as the vectors of the low chlorinated Tetra-CB and Tri-CB, denoting a different dehalogenation behavior with time according to the degree of chlorination. In this analysis, the vector representing the Family Dehalococcoidaceae was associated with vectors of taxa known for their sulfate-reducing and syntrophic activities, such as Desulfovibrionaceae (Genus *Desulfovibrio*) and Peptococcaceae (Genus *Desulfosporosinus*). This vector was opposed to the vectors of the highly chlorinated congeners (Hexa-CB and Hepta-CB) and was collinear with the Di-CB, an indication of the implication of this taxa in the dechlorination of highly chlorinated congeners. The vector of the Family Dehalococcoidaceae was opposite to the vector of the Family Geobacteraceae, as well as the one representing unclassified Desulfuromonadales and unclassified δ-Proteobacteria sequences, which can be statistically interpreted as a mechanism of mutual exclusion.

Contributions of the Dehalococcoidaceae ranged from a marginal 0.05% in SFM2 to 24.22% in SFM3. Deeper phylogenetic analysis of 1191 sequences retrieved from all SFMs and affiliated with Class Dehalococcoidia showed a dominance of sequences closely affiliated with *Dehalococcoides mccartyi* strains CBDB1 (878 sequences), BAV1 and CG5 (172 sequences), and 195 (78 sequences) (Figure SI-2). 63 sequences only shared more distant relationships with *Dehalogenimonas* sp. strain WBC-2. These obligate OHRB were considered as the main actors of the PCB dehalogenation in the SFMs without BES, in analogy with preceding studies (Bedard et al., 2007; Adrian et al., 2009; LaRoe et al., 2014; Wang et al., 2014). In contrast, members of the Class Dehalococcoidia were almost completely absent in BES-treated SFMs (Figure SI-5). One sequence only could be retrieved from SFM13 and one from SFM11, composing a marginal contribution of 0.04 and 0.02% of the respective communities. The sole sequence found in the BES-treated SFM13 shared 99% similarity with *D. mccartyi* strain CBDB1 (Figure SI-2). The other sequence affiliated with the Class Dehalococcoidia found in the BES-treated SFM11 showed a distant relationship with *D. mccartyi* strain BTF08 (87% similarity only).

Further insight into the putative role played by congener degraders was obtained using pairwise Pearson correlation coefficients, which were computed for PCB congener classes and bacterial Genera (Tables SI-6, SI-7). The results showed that a restricted number of taxa only displayed significant negative correlations (*p* < 0.05^*^) with PCB congener classes. Among SFMs without BES members of Genus Kosmotoga were correlated with the Penta-CB congener classes. Members of the Family Dehalococcoidaceae were not significantly correlated with a specific congener class, an indication for the implication of this taxon in the reduction of all classes of PCB congeners.

**Figure 2:**
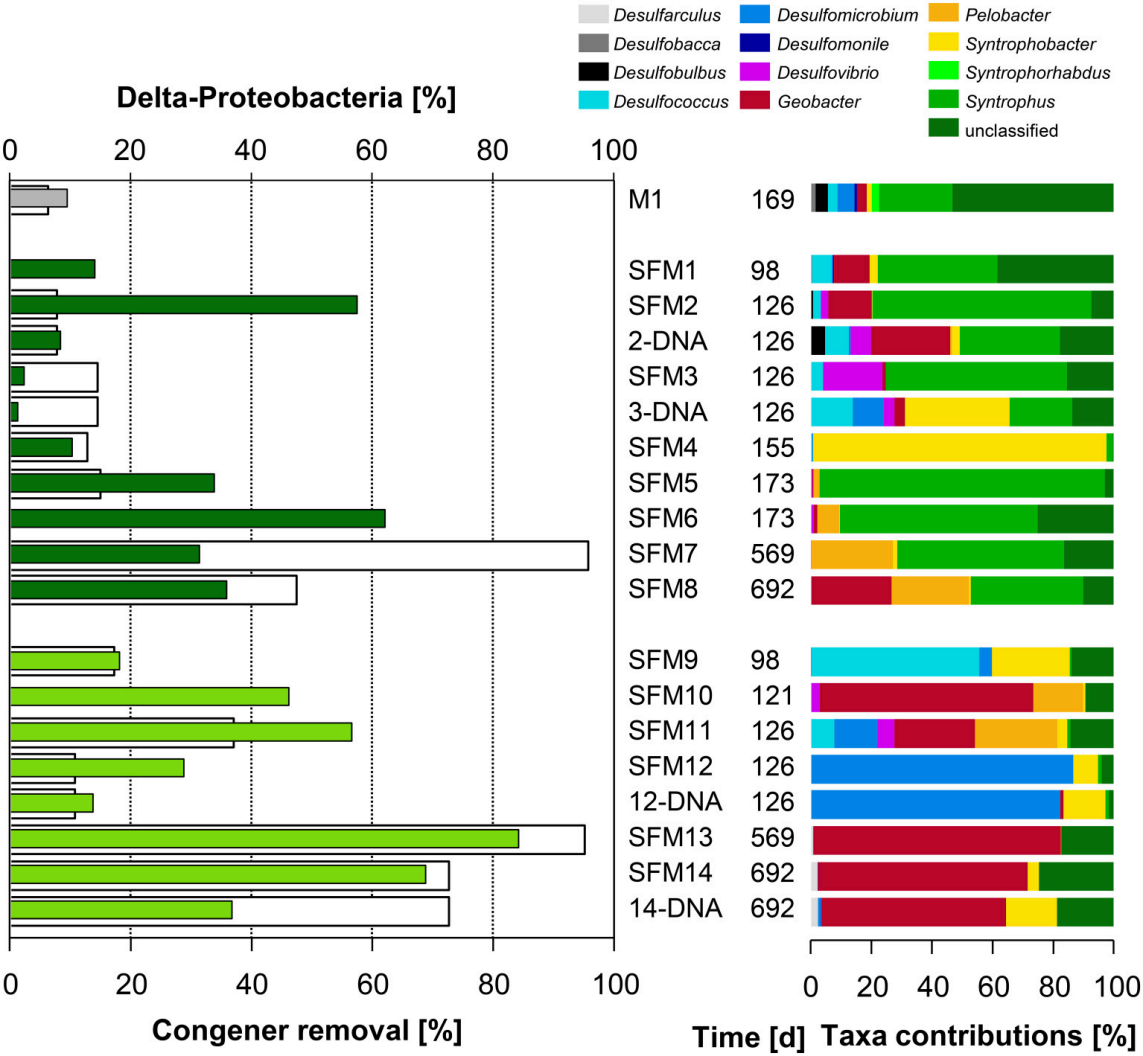
**Left:** total congener removal (in %, white bars) and relative contributions of sequences within the Class δ-Proteobacteria (in %, gray: initial sediment microcosms M1, dark green: no BES, light green: BES added). **Right:** relative contributions (in %) of Genera present within the Class.

The addition of BES promoted the development of the Class δ-Proteobacteria (Figure 2). Significant contributions of this Class were found in long-term microcosms (up to 84.2% in SFM13 and 68.8% in SFM14). Unclassified members of the Family Geobacteraceae showed strong and significant (*p* < 0.05^*^) negative correlations with the Penta-CB congener class (Table SI-7).

The Order Desulfomonadales is composed of iron-reducing bacteria of the Families Geobacteraceae (*Geobacter*) and Pelobacteraceae (*Pelobacter*). Whereas *Pelobacter* sp. was restricted to low relative abundances within the communities, the relative abundance of *Geobacter* sp. strongly increased with the addition of the BES, reaching up to 69.3% of all sequences found in SFM13. Deeper phylogenetic analysis using more than 6000 sequences from all SFMs showed the formation of a tight taxon, called “Strazske cluster” hereafter, sharing only 94% similarity with the closest relative *Geobacter* strain CdA-3 (Figure SI-3).

**Figure 3:**
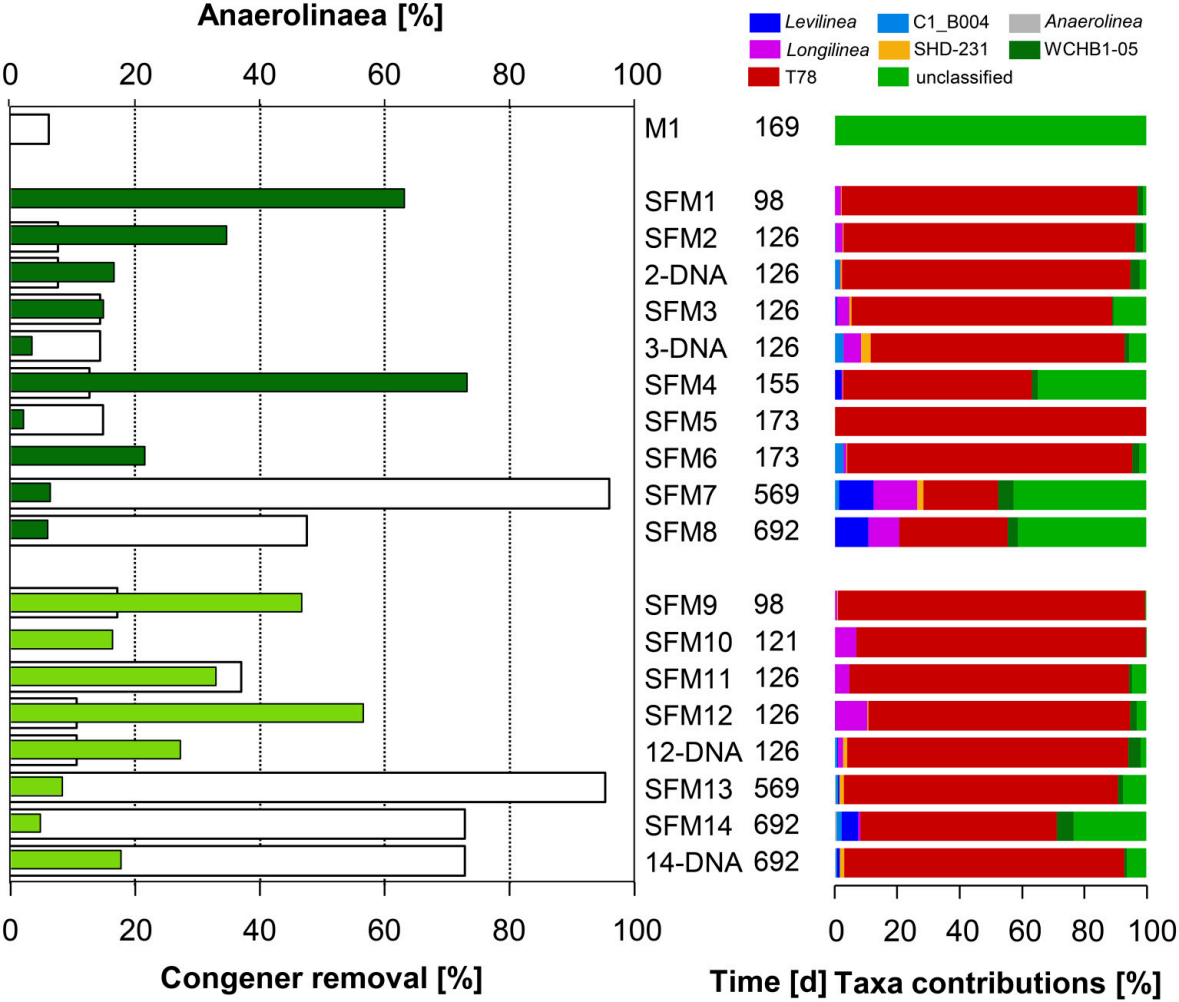
**Left:** total congener removal (in %, white bars) and relative contributions of sequences within the Class Anaerolinae (in %, gray: initial sediment microcosms M1, dark green: no BES, light green: BES added). **Right:** relative contributions (in %) of Genera present within the Class.

Among the Class δ-Proteobacteria, the Families of syntrophic bacteria Syntrophobacteraceae (including the Genus *Syntrophobacter*), Syntrophaceae (*Syntrophus* and *Desulfobacca*) and Desulfobulbaceae (*Desulfobulbus*) decreased in relative abundance in the presence of BES. Families related to sulfate-reduction, such as the Desulfomicrobiaceae (including the Genus *Desulfomicrobium* present in high proportions in SFM12), and in lesser proportions, Desulfovibrionaceae (*Desulfovibrio*) and Desulfarculaceae (*Desulfarculus*) were more abundant in the presence of BES (Figure 3). Sulfate reducers have been shown already to support the growth of OHRB, contributing indirectly to PCB dechlorination (May et al., 2008). Finally, MFA analysis pointed out the implication of two clades composed of unaffiliated sequences accompanying the Geobacteraceae vector (Figure 1). Phylogenetic analysis involving large fractions of unaffiliated sequences at the Desulfuromonadales or the δ-Proteobacteria phylogenetic levels showed the presence of distant clades sharing low similarities with the Genera *Geobacter/Pelobacter* and the syntrophic *Smithella*/*Syntrophus*, respectively (data not shown).

The Class Anaerolineae (Phylum *Chloroflexi*) was found in significant proportions in all SFMs (up to 73.2% in SFM4) (Figure 3) and was not significantly impacted by the addition of BES. Interestingly, the relative proportion of Anaerolineae in the original sediment microcosm M1 was very low (0.1%) and was composed mainly of unclassified organisms. In all SFMs, a large number of sequences were affiliated with Genus T78 (up to 98% in SFM9). Genus T78 is present in anaerobic digestion and biogas systems. Recent genomic analysis revealed that members likely metabolize alcohols and carbohydrates via syntrophic interactions. This clade was shown to be distantly related to cultured strains, having for closest representative *Longillinea arvoryzae* (Lamarche-Gagnon et al., 2015).

**Figure 4:**
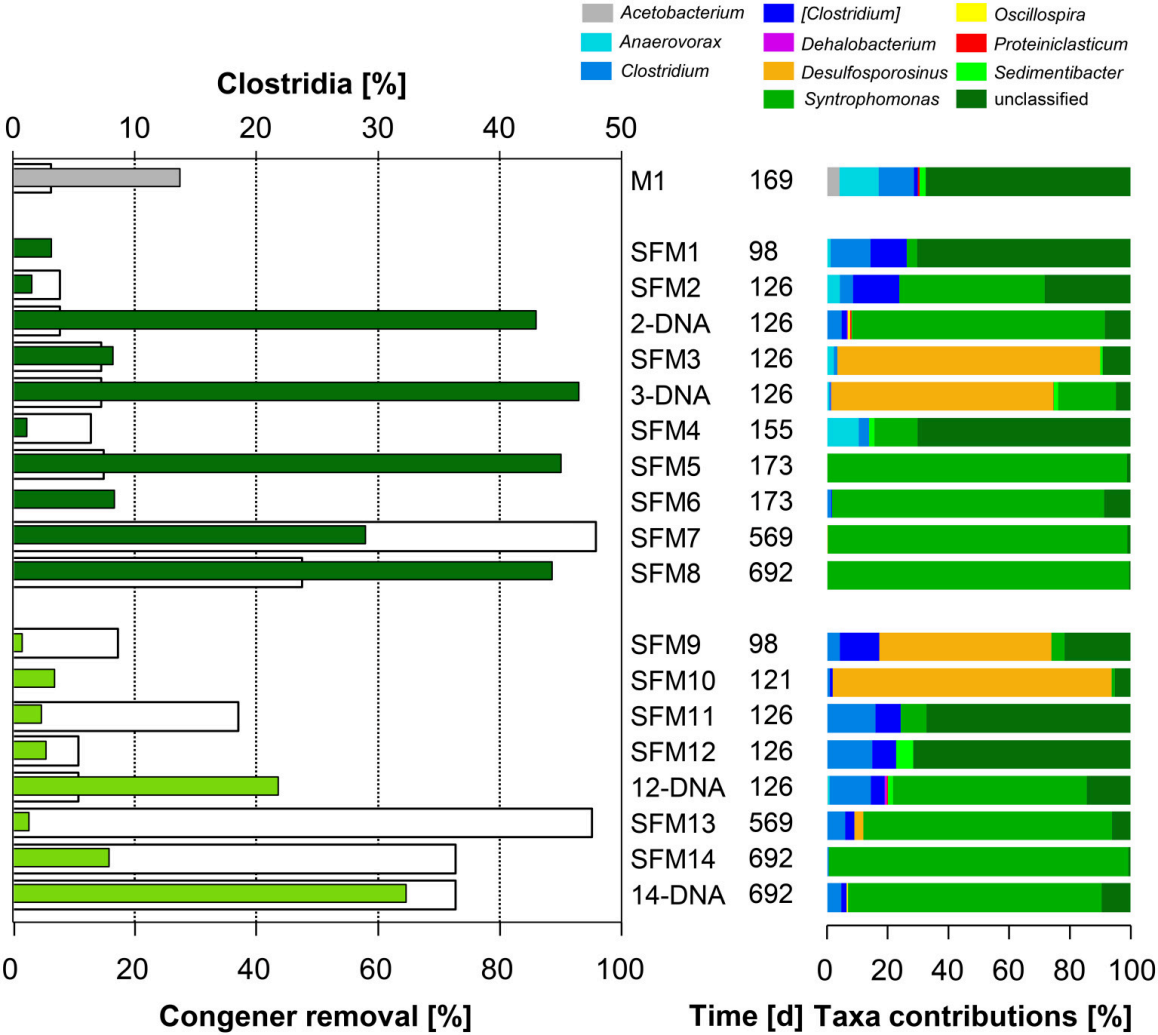
**Left:** total congener removal (in %, white bars) and relative contributions of sequences within the Class Clostridia (in %, gray: initial sediment microcosms M1, dark green: no BES, light green: BES added). **Right:** relative contributions (in %) of Genera present within the Class.

No OHRB belonging to the Firmicutes (such as *Dehalobacter* and *Desulfitobacterium*) was found in the SFMs. Contribution of this Phylum was restricted to the Class Clostridia only, and the respective Families Clostridiaceae, Peptostreptococcaceae, Peptococcaceae, and Syntrophomonadaceae (Figure 4). Vectors of these four Families were displayed in the MFA analysis (Figure 1) indicating their potential contribution to the congener dehalogenation in terms of fermentation and syntrophic interactions. The addition of BES had no significant impact on the distribution of the above mentioned Genera belonging to these Families. Contributions of the Clostridiaceae (*Clostridium*), Peptostreptococcaceae ([*Clostridium*]) and Peptococcaceae (*Desulfosporosinus*) decreased in later SFMs. The Genus *Sedimentibacter* (Family XI) did not contribute significantly to the communities (SFM3 and SFM12 only) despite former studies revealing its predominant role in syntrophic interactions with OHRB (Oh et al., 2008). Interestingly, the relative contributions of the *Firmicutes* were found inversely proportional to the Anaerolineae (Phylum *Chloroflexi*) in the SFMs without BES. *Firmicutes* composed the largest taxa in the SFM5 (46.96%) and SFM8 (45.90%) for instance, in which Anaerolineae contributed marginally with a mere 2.22 and 6.22% only. Inversely, Anaerolineae were dominant in SFM4 (74.06%) and SFM1 (65.53%) with a contribution of the *Firmicutes* reaching only 1.27 and 3.45% respectively. Large proportions of Syntrophomonadaceae (*Syntrophomonas*). *Syntrophomonas* sp. were present, up to 46.0% in SFM5. Dechlorination activity in presence of the *Syntrophomonas* sp. was demonstrated already in co-cultures with OHRB (Hug, 2012; Mao et al., 2015). Finally, members of these Families composed significantly high proportions of the communities when total DNA was examined, indicating that large amounts of cells were present, albeit showing very low biological activities. Other Phyla (such as *Bacteroidetes, Planctomycetes, Synergistetes, Thermotogae*, and WWE1) composing up to 20% of the communities found in the SFMs (Table SI-4; Figure SI-4) were not impacted significantly by the addition of BES and showed no significant correlation with the congener classes. The vector of the Family S-70 (Phylum *Planctomycetes*) showed the only occurrence, with collinearity with syntrophs present within the Families Syntrophomonadaceae and Pelobacteraceae in the MFA analysis (Figure 1).

Finally, the archaeal community present in untreated SFMs was composed exclusively of members of the Phylum *Euryarchaeota*, with the dominant abundance (up to 96.6% in SFM6) of a unique member of the Methanosarcinales (Genus *Methanosaeta*) known for its acetoclastic methanogenic activities. Methanobacteriales, which are generally hydrogenotrophic, and members of the Order Thermoplasmata, a poorly characterized taxon putatively known as methylotrophic methanogens (Borrel et al., 2013), were present in low relative proportions only. The addition of BES did not result in the complete inhibition of *Archaea*, as measured after 126 days of incubation (Table SI-5). GC-FID analysis of the gas phase of BES-treated SFMs showed the absence of methane and the presence of large amounts of ethene after 60 days of incubation (data not shown). Ethene has earlier been shown to be a BES transformation product of methanogenic *Archaea* (Holliger et al., 1992). The archaeal communities in the BES-treated SFMs were almost identical as the ones present in untreated SFMs (Table SI-5). They were however enriched with 3.0–10.1% (SFM11 and SFM12, respectively) of sequences affiliated with the Class MCG (for Miscellaneous Crenarchaeota Group) within the *Crenarchaeota*. This taxon has recently been shown to be a predominant archaeal group in anoxic environment, known for their role in the dehalogenation of aromatic compounds (Meng et al., 2014).

## Discussion

The goals of the present study were to explore further the OHRB guild involved in the dehalogenation process observed in anaerobic cultivations of primary sediments from the PCB-contaminated efflux channel in Eastern Slovakia (Praveckova et al., 2015). SFMs supplemented with Delor 103 were studied by coupling of massive sequencing of the enriched communities with numerical statistical analysis as a strategy for the identification of actors involved in the dehalogenation. Selecting a cultivation medium was prerequisite for long-term cultivation. The *Dehalobacter* culture medium provided favorable conditions for microbial consortia, with the initial addition of a large selection of vitamins and trace elements, including cobalamin, the key cofactor of reductive dehalogenases. A slow release of hydrogen was obtained with the addition of fermentable substrates (ethanol, butyrate, propionate, and acetate) on a regular basis that are degraded by syntrophic interactions favoring such reductive dechlorination over other hydrogenotrophic processes (Fennell et al., 1997).

Sequences affiliated with members of the Class Dehalococcoidia showed strong similarities with strains known for their capacity to degrade PCB congeners, such as *D. mccartyi* strains CG4 and CBDB1 (Adrian et al., 2009; Wang et al., 2014). The dehalogenation of the congeners measured in the SFMs, from Di-CB to Hepta-CB, was interpreted as a result of the activity of these strains, according to recent studies. Wang et al. characterized three *Dehalococcoides* strains that were involved in the dehalogenation of PCB congeners, and showed the presence of unique enzymes capable of both perchlorethylene and PCB congener reduction (Wang et al., 2014). Addition of BES in the SFMs induced drastic changes, such as the almost complete inhibition of members of the Family Dehalococcoidia. BES is a structural analog of the coenzyme M (Co-M) which is synthetized by methanogenic *Archaea* as a methyl group carrier during methanogenesis (Gunsalus et al., 1978). However, BES is not only a potent inhibitor of the methanogenesis as it was shown to also inhibit the degradation of short-chain aliphatic alkenes which are processed by syntrophic organisms in mutualistic interactions (Boyd et al., 2006). Obligate OHRB such as *Dehalococcoides* sp. live in syntrophic interactions with H_2_-producing populations and interdependence between OHRB and their syntrophic partners has an impact on the dehalogenation process (Becker et al., 2005). Hiraishi showed that different members of the *Chloroflexi* phylum exhibited a limited range of PCB congener dechlorination, which strongly suggested a complex mechanism and the possible significance of synergistic interactions with the contribution of other phyla to the transformation of these compounds (Hiraishi, 2008). Whereas BES apparently had no impact on the abundance of members of the Class Anaerolineae (Phylum *Chloroflexi*), it impacted strongly other syntrophs, such as the Syntrophaceae and Cloacamonaceae. Other Phyla were also absent in BES-treated SFMs, such as OP3 (Obsidian Pool 3) and koll11, whose members are significantly always co-occurring with Dehalococcoidia in this study. OP3 organisms, recently named phylum *Omnitrophica* (Rinke et al., 2013), and belonging to the *Planctomycetes*/*Verrucomicrobia*/*Chlamydiae* (PVC) superphylum (Fuerst, 2013) are reported for diverse habitats including anoxic sediments such as flooded paddy soils and marine sediments (Glöckner et al., 2010).

Despite the absence of Dehalococcoidia, high rates of congener dehalogenation were measured, an indication for an activity carried out by other OHRB guild members. BES addition resulted in the development of members of the Family Geobacteraceae. Members of this Family are metabolically versatile, including metal- and sulfate-reducers. The Genus *Geobacter* has been shown to use a broad range of electron acceptors including nitrate, fumarate, Fe(III), malate, Mn(IV), U(VI), and elemental sulfur (Sung et al., 2006). *Geobacter thiogenes* strain K and *Geobacter lovleyi* strain SZ are the unique representatives of the Family Geobacteraceae capable of coupling the reduction of organohalides to energy conservation and growth. Furthermore, *G. lovleyi* is capable of de novo cobalamin synthesis, an essential component of reductive dehalogenases (Hug, 2012). Both strains were however present in marginal numbers in the present study. Recently, a detailed analysis of the genome of *G. lovleyi* revealed that lateral gene acquisition was the source of the capability to use chlorinated compounds as electron acceptors (Wagner et al., 2012). Genes related to PCE reduction showed the highest homology with reductases present in the *Firmicutes Dehalobacter restrictus* and *Desulfitobacterium hafniense*. Sequences that were associated with the Geobacteraceae formed a homogenous “Strazske” cluster that shared ca. 94% similarity with *G. lovleyi* only, indicating the presence of yet unknown taxon. Indirect evidence, such as the augmentation of the relative proportions of the taxa in presence of BES, the length and the direction of the corresponding vector in the MFA analysis, and the statistically significant computations using pairwise Pearson correlation coefficients suggested that members of the Strazske cluster could be involved in the dechlorination of PCB congeners. From what precedes, lateral gene acquisition of new respiratory capacities could have been the source of the dehalogenating activity. The acquisition of genes encoding reductive dehalogenases catalyzing chlorine removal from low chlorinated congeners could make this *Geobacteraceae* cluster a putative candidate for the dehalogenation of PCB congeners. This hypothesis is reinforced further by the absence of any other facultative OHRB in BES-treated SFMs, such as the Genera *Dehalobacter, Desulfitobacterium* (*Firmicutes*), and *Sulfurospirillum* (ε-*Proteobacteria*) *Shewanella sediminis* (δ-*Proteobacteria*). Interestingly also, a large dominance of sequences affiliated with the aceticlastic methanogen *Methanosaeta* sp. was found in all SFMs. *Methanosaeta* in co-culture with *Geobacter* was proved to support strongly the growth of the latter via direct interspecies electron transfer (DIET) process (Rotaru et al., 2015). In conclusion, this study showed for the first time that members of the Geobacteraceae, forming a tight cluster remotely related to the Genus *Geobacter*, could be involved in the dechlorination of PCB congeners. Further studies on the role played by this cluster are needed so as to link their presence with the dehalogenation patterns observed on the polluted site of Strazske.

## Author contributions

MP carried out research, data handling and analysis and wrote the manuscript. PR contributed to the design of the experiments, supervised the analysis and contributed to data mining and the writing of the manuscript. MB and CH contributed to the design of the experiments and the manuscript edition. FD contributed to the analysis of the PCB congeners and to the manuscript edition.

### Conflict of interest statement

The authors declare that the research was conducted in the absence of any commercial or financial relationships that could be construed as a potential conflict of interest.
