# Supplementary material for: Indirect Evidence Link PCB Dehalogenation with Geobacteraceae in Anaerobic Sediment-Free Microcosms
Source: Front Microbiol. 2016 Jun 16;7:933. doi: 10.3389/fmicb.2016.00933 (PMC4909783; doi:10.3389/fmicb.2016.00933)
Supplement: Supplementary file 1 [file Table1.docx]

# Supplementary information

# **Indirect evidence link PCB dehalogenation with Geobacteraceae in anaerobic sediment-free microcosms**

Martina Praveckova^1, 2^, Maria Brennerova^1^, Felippe de Alencastro^4^, Christof Holliger^3^ and Pierre Rossi^4*^

**AFFILIATIONS:**

1. Laboratory of Molecular Genetics of Bacteria, Institute of Microbiology, Academy of Sciences of the Czech Republic, Prague, Czech Republic

2. Charles University, Faculty of Science, Prague, Czech Republic

3. Central Environmental Laboratory, School of Architecture, Civil and Environmental Engineering, Ecole Polytechnique Fédérale de Lausanne, Lausanne, Switzerland

4. Laboratory for Environmental Biotechnology, School of Architecture, Civil and Environmental Engineering, Ecole Polytechnique Fédérale de Lausanne, Lausanne, Switzerland

**Table SI-1**: Concentrations of the congener (in mg/L) measured in the SFMs.

**Table SI-2**: Inference statistics computed for the communities present in the SFMs. Bacterial communities were analyzed from total RNA extracts, to the exception of the communities mentioned with “#-DNA” which were obtained from total DNA.

After quality control steps (such as denoising and the removal of putative chimeras), subsequent analyses were carried out on a total of 49686 sequences, ranging from 1193 (sample M1) to 6282 sequences (SFM7).

OTU numbers did not evolved significantly between the original sample M1 and the SFMs. OTU numbers reached a maximum of 669 in SFM11, and a minimum in SFM5, when measured in total RNA. For DNA samples, the values reached 530 (20-DNA) and 159 (6-DNA) respectively.

Total amounts of different Genera found in the sediment microcosm M1 was significantly higher than the ones detected in all SFMs. These discrepancy was reflected equally in the values obtained for the different indices. Diversity indices showed a strong decrease in terms of diversity and evenness in the SFMs in comparison with the original sediment microcosm M1. ACE and Chao1 showed ca. 2000 species in M1 and this was reduced to 200-700 in DNA SFMs. ACE estimator of species richness showed that the richest communities were SFM11 (1366) and SFM1 (1154), while the poorest microcosm was established SFM5 (293). Chao1 index confirmed the data measured by ACE method.

Shannon H´ values showed the highest diversity in SFM3 (2.44) and SFM11 (2.46). On the other hand, significantly lower diversity was detected in SFM13 (1.29, resp.1.99). In terms of the evenness, Shannon and Pielou’s evenness indexes showed between 0.07, resp. 0.33 (SFM13) to 0.21, resp.0.61 (SFM11).

Fisher´s alpha sample size independent coefficient showed that the richest community was SFM12 while the lowest value was calculated for SFM8. In general, the most abundant, diverse and even sample was SFM11 and lowest values were calculated for SFM13. There was no impact of BES addition on the apparent richness, diversity and evenness of the communities.

**Figure SI-1a**: Rarefaction analysis (3% clustering level) carried out on high-quality sequences based on total RNA extracted from the SFMs, treated with BES (left pane) or without (right pane) using ESPRIT-Tree.

**Figure SI-1b**: Rarefaction analysis (3% clustering level) carried out on high-quality sequences based on total DNA extracted from the SFMs, treated with BES (SFM12 and 14) or without (SFM2 and 3) using ESPRIT-Tree.

**Table SI-3**: Relative contributions (in %) of all phyla detected in the SFMs as well as in the sediment microcosm M1.

**Table SI-4**: Relative contributions (in %) of major Phyla, with corresponding Classes and Families present in the SFMs and in the sediment microcosm M1.


**Table SI-4**: continued

**Table SI-5**: Relative contributions (in %) of major Phyla, Classes, Orders and Families of the Archaea composing the communities present in four SFMs.

**Table SI-6**: Pearson correlations computed between bacterial Genus and congener classes measured in the SFMs (no BES). Significant correlations (p<0.05*) are indicated with black bordering.

**Table SI-7**: Pearson correlations computed between bacterial Genus and congener classes measured in the BES-treated SFMs. Significant correlations (p<0.05*) are indicated with black bordering.

11 reads **SFM6**

2 reads **SFM3**

**CP011392 *Dehalogenimonas* *sp.* WBC-2**

**SFM11** HDXKKXU02EHYWT

1 read **SFM3**; 47 reads **SFM6**; 1 read **SFM1**

**CP000688 *D. mccartyi* strain BAV1**

**CP001827 *D. mccartyi* strain VS**

**CP006951 *D. mccartyi* strain CG5**

**CP006950 *D. mccartyi* strain CG4**

**CP006949 *D. mccartyi* strain CG1**

157 reads **SFM3**; 4 reads **SFM8**; 9 reads **SFM1**; 2 reads **SFM7**

**CP000027 *D. mccartyi* strain 195**

**SFM3** HDXKKXU02DHA7C

**SFM3** HDXKKXU02DM4RG

12 reads **SFM3**; 2 reads **SFM5**

21 reads **SFM3**, 1 read **SFM1**, 1 read **SFM7**

**SFM3** HDXKKXU02DORKM

**SFM3** HDXKKXU02DDVXQ

**SFM8** H2N7ATJ02D7RFV

**SFM8** H2N7ATJ02D9A3Q

**SFM3** HDXKKXU02EE371

**SFM3** HDXKKXU02EYUPJ

**SFM3** HDXKKXU02DUC7U

**SFM3** HDXKKXU02DQQ23

14 reads **SFM3**, 5 reads **SFM1**

**SFM4** HDXKKXU02D5WQS

**SFM4** HDXKKXU02D4CHV

10 reads **SFM3**

**AJ965256 *D. mccartyi* strain CBDB1**

762 reads **SFM3**; 10 reads **SFM5**

32 reads **SFM8**, 11 reads **SFM4**

54 reads **SFM1**, 8 reads **SFM7**

1 read **SFM13**

**HM241729 *Dehalococcoides sp.* ANAS1**

**NR 074337 *Dehalogenimonas lykanthroporepellens*** **BL-DC-9**

**AP010960 *Escherichia coli***

0.05

**Figure SI-2**: Neighbour-joining phylogenetic tree (Maximum composite likehood) of sequences affiliated with the Class Dehalococcoidia. All sequences (1191) obtained from all SFMs (red: BES-treated SFMs; blue: no BES) are displayed.

**SFM5** HYLX8LQ02FQLZS

**SFM8** H2N7ATJ02EHY7D

**SFM8** H2N7ATJ02EKUIY

**SFM5** HYLX8LQ02J4CPQ

**SFM8** H2N7ATJ02EKL9C

**SFM8** H2N7ATJ02EMFF1

**SFM6** HDXKKXU02EDOGL

**SFM8** H2N7ATJ02EJ7KD

**SFM7** H22H9YA03GCJUR

**SFM1** HDXKKXU02D3WEP

**SFM1** HDXKKXU02D02NA

**SFM1** HDXKKXU02D0WZ3

**SFM1** HDXKKXU02CYTX2

**SFM3** HDXKKXU02DHAWO

**SFM12** HDXKKXU02DJRHO

**SFM10** HYLX8LQ02JV98X

**SFM10** H22H9YA03FLE1L

**SFM10** H22H9YA03GV4BV

**SFM10** H22H9YA03G15FA

**SFM2** H72HL4A01A6EAL

**SFM2** H72HL4A01A5VTU

**SFM10** H22H9YA03G0IFK

**SFM11** HDXKKXU02EKXKF

**SFM2** H72HL4A01A4SSA

**SFM2 H72HL4A01A6COE**

**SFM7** H22H9YA03G1Z7F

**SFM14** HYLX8LQ02JEY3Z

**SFM7** H22H9YA03HC68H

**SFM2** H72HL4A01A60G2

**SFM2** H72HL4A01A6A2S

**SFM11** HDXKKXU02EL6T4

**SFM14** HYLX8LQ02JF4N8

**SFM13** H22H9YA03G6OQM

**SFM14** HYLX8LQ02JERH9

**SFM7** H22H9YA03GFXJ7

**SFM7** H22H9YA03FYM0Q

**SFM13** H22H9YA03G5Q8D

**SFM7** HYLX8LQ02JSIVV

**SFM6** HDXKKXU02EC74I

**SFM13** H22H9YA03GDCC0

**SFM14** HYLX8LQ02JDQ6L

**SFM6** HDXKKXU02DKQPH

**SFM13** H22H9YA03GACU9

**SFM7** H22H9YA03G8TCA

**SFM13** H22H9YA03G65OX

**SFM11** HDXKKXU02EL2F4

**Y19191 Geobacter sp. strain CdA 3**

**JF342225 Uncultured bacterium clone 432MICCbiofilm**

**NR 074975 Pelobacter propionicus strain 2379**

**JQ088365 Uncultured bacterium clone M16-9-B01**

**X70955 Pelobacter acetylenicus**

**EF059536 Geobacteraceae bacterium JN18_V95_J**

**HM749859 Uncultured bacterium clone 2**

**EF507967 Uncultured bacterium clone FRC-A2_434**

**NR 074979 Geobacter lovleyi strain SZ**

**JN982204 Geobacter lovleyi strain Geo7.1A**

**HQ655446 Uncultured bacterium clone EG7N**

**AB231802 Anaerobic syntrophic bacterium NE23-3**

**AP010960 Escherichia coli**

0.5

**Figure SI-3**: Neighbour-joining phylogenetic tree (Maximum composite likehood) of sequences affiliated with the Family Geobacteraceae showing sequences randomly selected among more than 6000 sequences obtained from all SFMs (red: BES-treated SFMs; blue: no BES) .

**Figure SI-4**: Left: relative contributions (in %) of sequences affiliated with diverse minor phyla (grey: initial sediment microcosms M1, dark green: SFMs, light green BES-SFMs) as a function of the total congener removal (in %, white). Right: relative contributions (in %) of the phyla.

**Figure SI-5:** Left: relative contributions (in %) of sequences affiliated with the Class Dehalococcoidia (grey: initial sediment microcosms M1, dark green: SFMs, light green BES-SFMs) as a function of the total congener removal (in %, white). Right: relative contributions (in %) of the Genera.
